# Cerebral blood flow abnormalities with central sparing on arterial spin labeling in mild encephalopathy associated with excitotoxicity: a case report

**DOI:** 10.1186/s12883-022-02942-5

**Published:** 2022-11-02

**Authors:** Yuki Nakajima, Shinya Kobayashi, Hideki Tanoue, Sayaka Ishihara, Ayako Kamiya, Nanako Kawata, Mari Asakura, Daichi Suzuki, Natsuko Obana, Kenta Hayashi, Takahiro Kawaguchi, Masahiro Noda, Kunihiro Oba, Tatsuo Katori, Tsutomu Kageyama, Masashi Ogasawara

**Affiliations:** 1 Department of Pediatrics, Showa General Hospital, Tokyo, Japan; 2 Center for Emergency Preparedness and Response, National Institute of Infectious Diseases, Tokyo, Japan; 3 Department of Neuromuscular Research, National Institute of Neuroscience, National Center of Neurology and Psychiatry (NCNP), 4-1-1 Ogawahigashi, Kodaira, Tokyo, Japan

**Keywords:** Acute encephalopathy with biphasic seizures and late reduced diffusion, Mild encephalopathy associated with excitotoxicity, Arterial spin labeling, Magnetic resonance imaging, Cerebral blood flow, Glutamate, Glutamine, Early diagnosis

## Abstract

**Background:**

Acute encephalopathy with biphasic seizures and late reduced diffusion (AESD) and mild encephalopathy associated with excitotoxicity (MEEX) are the most frequent acute encephalopathies in pediatric patients in Japan. AESD typically presents with biphasic seizures and delayed reduced diffusion in the subcortical area, called bright tree appearance (BTA), on radiological examination. In patients with AESD, arterial spin labeling (ASL) shows decreased cerebral blood flow (CBF) in the hyperacute stage and increased CBF in the acute stage, suggesting the usefulness of ASL for the early diagnosis of AESD. Additionally, proton magnetic resonance spectroscopy (MRS) shows elevated glutamate (Glu) and glutamine (Gln) in AESD. MEEX is a group of mild encephalopathies with transient elevation of Gln on MRS similar to that in AESD; however, MEEX does not include any clinical biphasic course or abnormalities, including BTA on diffusion-weighted imaging. Although the usefulness of ASL for AESD has been reported, there are no reports for patients with MEEX. In this study, we report our experience with a 4-year-old girl diagnosed with MEEX who showed unique findings on ASL.

**Case presentation:**

The patient was a 4-year-old girl admitted to the emergency room with febrile status epilepticus. Considering the possibility of AESD, vitamin therapy was initiated. ASL-MR imaging (MRI) of the brain performed on the second day showed increased blood flow in the frontal, temporal, and occipital regions with spared central sulcus, which indicated AESD with central sparing. The patient was diagnosed with AESD, and the treatment included pulse steroid therapy and immunoglobulin therapy from day 3. The patient remained mildly unconscious but gradually became conscious by day 7 with no seizures. Brain MRI performed on day 8 did not show any characteristic AESD findings, such as BTA. Furthermore, MRS showed elevated Gln, which, along with the clinical course, led to the diagnosis of MEEX. The patient was discharged on day 16 without obvious sequelae.

**Conclusions:**

ASL may be useful in the early diagnosis of MEEX as well as AESD, facilitating early intervention.

## Background

Acute encephalopathy with biphasic seizures and late reduced diffusion (AESD) is the most frequent subtype of pediatric encephalopathy in Japan, with an estimated 100–200 cases per year [1]. AESD is characterized by a biphasic clinical course and delayed imaging findings [2, 3]. The first symptom of AESD is a prolonged febrile seizure (early seizure), after which the disturbance of consciousness tends to improve (almost resolved in 20–30% of cases) [4]. However, the seizures recur within 4–6 days (late seizures), and the disturbance of consciousness worsens [4]. After a second seizure cluster, aphasia, loss of spontaneity, and stereotypic movements become apparent [2]. Magnetic resonance imaging (MRI) shows no abnormality between days 1 and 2, but it shows restricted diffusion—high signal intensity on diffusion-weighted imaging (DWI) and low signal intensity on the apparent diffusion coefficient (ADC) map—of subcortical white matter usually with bifrontal predominance, which is called bright tree appearance (BTA) during days 3 to 9 [3, 5]. Interestingly, BTA, in most cases, is not observed in the pre- and post-central gyri, which is called “central sparing” [2, 6]. Since the perirolandic regions of infants aged between 1 and 2 are considered resistant to ischemic injury [7], the presumed pathophysiological implication of central sparing is thought to be ischemic damage [5]. In addition, MR spectroscopy (MRS) in patients with AESD show elevated glutamate (Glu) from days 1 to 4, followed by elevated glutamine (Gln) from days 4 to 12 [8].

Mild encephalopathy associated with excitotoxicity (MEEX) is characterized by impaired consciousness lasting > 24 h with a prolonged febrile seizure but without late seizures and BTA on MRI. Similar to AESD, MEEX also shows Glu elevation followed by subacute Gln elevation on MRS. Therefore, AESD and MEEX are considered part of the same disease spectrum characterized by excitotoxicity caused by the Glu/Gln complex [9].

Arterial spin labeling (ASL) is a noninvasive MR cerebral blood flow (CBF) imaging method that does not use a contrast agent [10]. ASL in patients with AESD shows decreased CBF in the hyperacute phase (8–22 h after early seizures) but increased CBF in the acute phase (within 24 h after late seizures or on days 3–5 after early seizures) [6, 11]. The usefulness of ASL for AESD has been reported; however, it has not been confirmed in patients with MEEX [6, 11, 12]. In this study, we report our experience of a case involving a 4-year-old girl diagnosed with MEEX, which demonstrated unique findings on ASL.

## Case presentation

**Fig. 1:**
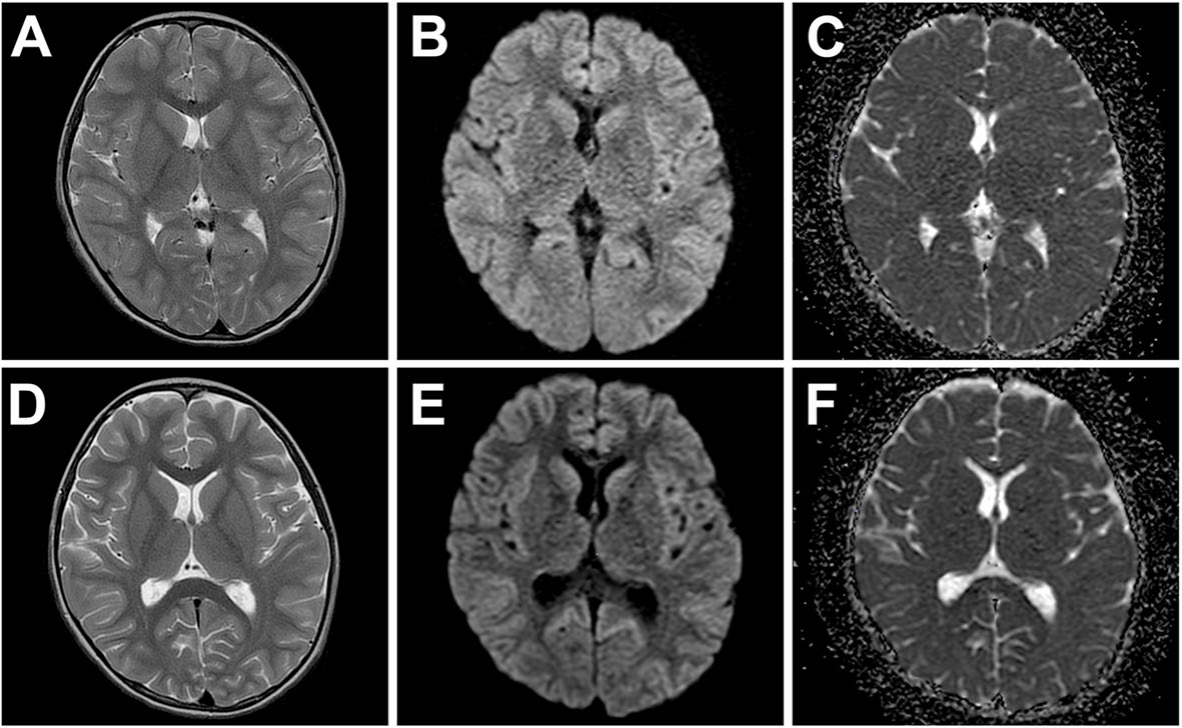
Brain MRI findings of the patient. Brain MRI images taken on the second (**A**, **B**, **C**) and eighth (**D**, **E**, **F**) days, where T2-weighted imaging (**A**, **D**), DWI (**B**, **E**), and ADC map (**C**, **F**). **D**, **E**, **F** Brain MRI on the eighth day shows no abnormalities except for mild cerebral atrophy

**Fig. 2:**
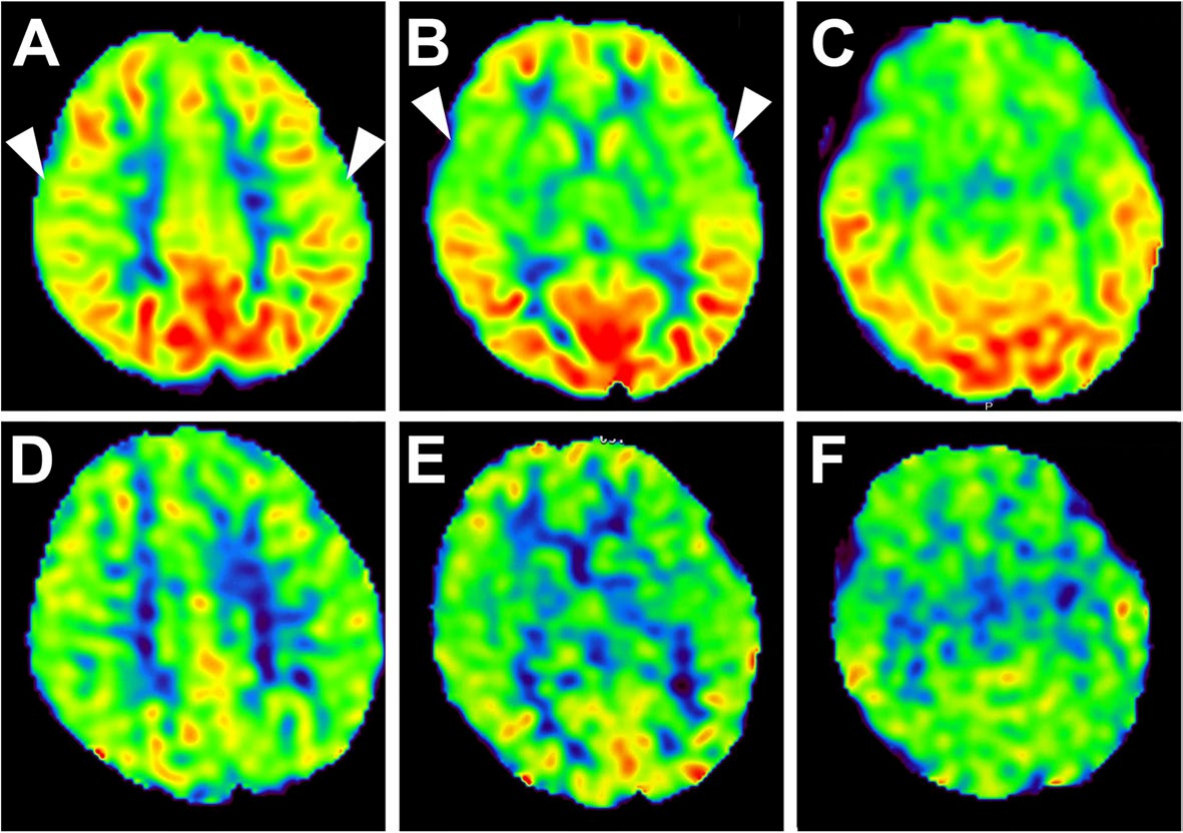
ASL of the patient. ASL images taken on the second (**A**–**C**) and eighth (**D**–**F**) days. **A**–**C** ASL shows increased blood flow in the frontal and occipital regions, sparing the central sulcus (arrowheads). **D**–**F** ASL shows no apparent abnormalities on the eighth day

**Fig. 3:**
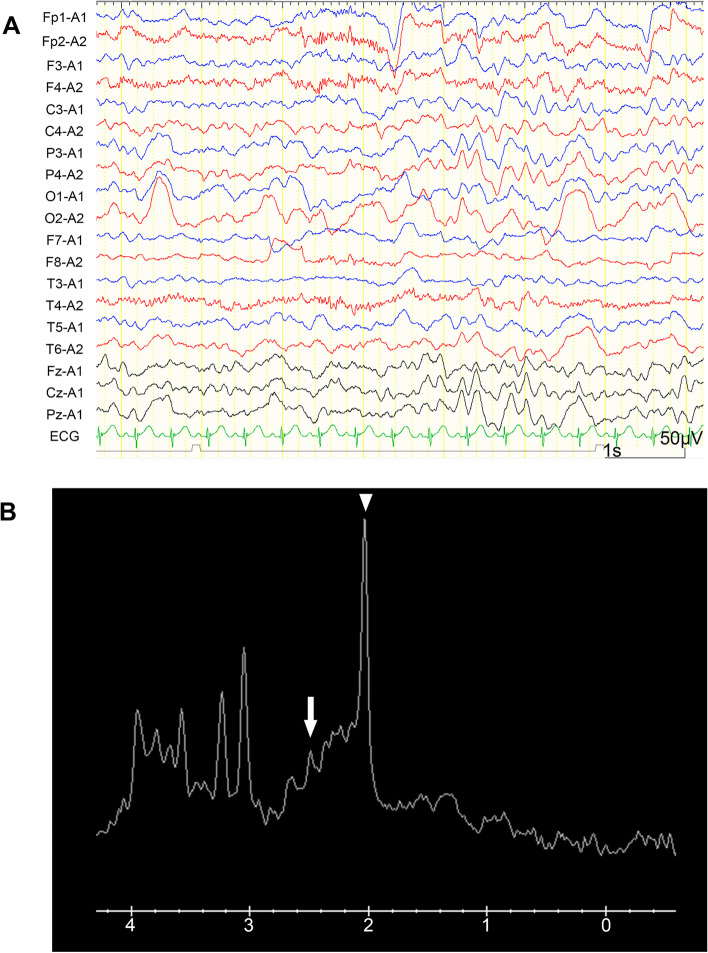
EEG and MRS findings of the patient. **A** Awake EEG shows high-voltage slow waves, especially in the right occipital lobe on the third day. **B** MRS image taken on the eighth day. The white arrowhead indicates N-acetyl-aspartate. The white arrow indicates glutamine (Gln)

A 4-year-old girl with no significant medical history was brought to the emergency department with febrile status epilepticus. Approximately 10 h after the onset of fever, generalized tonic–clonic convulsions appeared, lasting approximately 50 min. After the generalized seizures subsided, clonic convulsions of the left upper extremity continued in clusters. Midazolam, fosphenytoin sodium hydrate, and levetiracetam were administered. After the convulsions sudsided, in about 90 min, the patient remained mildly unconscious; therefore, head computed tomography and cerebrospinal fluid examination were performed, but no abnormalities were found. Human parainfluenza virus type 3 and human rhinovirus type C were co-detected using FilmArray® Respiratory 2.1 panel and in-house reverse transcription loop-mediated isothermal amplification assays from nasopharyngeal specimen, and they were thought to be the source of the fever. Because of the possibility of AESD, vitamin therapy (vitamin B1 100 mg/day, vitamin B6 20 mg/kg/day, and L-carnitine 30 mg/kg/day) was administered on the first day [13]. Although brain MRI including T1, T2, DWI, and ADC map on the second day (33 h after early seizure) showed no abnormalities (Fig. 1A–C), ASL showed increased blood flow in the frontal, temporal, and occipital regions with spared central sulcus, similar to the central sparing of AESD (Fig. 2A–C). In addition, electroencephalogram (EEG) showed high-voltage slow waves, especially in the right occipital region (Fig. 3A). The patient was diagnosed with AESD because of the prolonged disturbance of consciousness and ASL results at 33 h that pointed to a diagnosis of AESD in the acute stage. Therefore, she was treated with pulse steroid therapy and immunoglobulin therapy in addition to vitamin therapy from the third day. The patient remained mildly unconscious, but she gradually became conscious by the seventh day, and no late seizures occurred. A brain MRI performed on the eighth day did not show BTA; however, the cerebrum was generally and mildly atrophic (Fig. 1D–F), and this could possibly have been caused by pulse steroid therapy. Additionally, ASL performed on the eighth day showed no apparent abnormal CBF distribution (Fig. 2D–F). Furthermore, Gln elevation was observed on MRS on the eighth day (Fig. 3B). This finding together with the clinical course led to a diagnosis of MEEX. The vitamin cocktail treatment was completed after a total of 10 days. Although EEG still showed slow waves in the occipital lobe, there were no problems in motor function, conversation, or the state of consciousness. The patient was discharged on the sixteenth day without obvious sequelae.

## Discussion and conclusions

In this study, the MRI on the second day (33 h after early seizure) showed abnormal blood flow distribution on ASL, which led us to suspect AESD and to commence early treatment, resulting in discharge without sequelae. Although several scoring models for AESD and MEEX have been advocated, there are currently no validated biomarkers in the early phase, making it difficult to distinguish between AESD, MEEX, and early complex febrile seizures [4, 6]. Previous reports have shown that blood flow measurement using ASL can be effective in identifying seizures [10]. It is also known that in the first 4 h after a seizure, there is evidence of focal hypoperfusion [14, 15]. To date, there have been a few reports on the utility of ASL in patients with AESD [6, 11]. Most patients with AESD show decreased CBF in the hyperacute phase (8–22 h after first seizure) and increased CBF in the acute phase, suggesting that ASL may be useful for the early diagnosis of AESD but should be performed > 8 h after the onset of early seizures to exclude the possibility of hypoperfusion due to seizures [6, 11]. Conversely, ASL in patients with complex febrile seizures shows variable blood flow patterns without central sparing [16]. In our case, the MRI performed 33 h after the first seizure showed increased blood flow in the frontal, temporal, and occipital regions with spared central sulcus, which was similar to that observed in patients with AESD in the acute phase (Table 1).

**Table 1:** Overview of demographic data, clinical symptoms, and imaging

	Demographic data			Clinical symptoms			Imaging			Reference
Diseases	Age	Sex	Pathogens	1st seizure (SE)	IC > 24 h	2nd seizure	MRI	MRS	ASL	
CFS	6 months– 6 years	M > F	Rhino, Adeno, Parainflu, Flu	+	-	-	NA	NA	Variable	[16, 17]
MEEX	5 months– 2 years	UK	Rota, HHV-6 Flu	+	+	-	NA	Day 3 Glu↑ Day 3–8 Gln↑	UK	[4, 9]
AESD	10 months– 4 years	M = F	Flu, HHV-6/7 Rota, RSV	+	+	+	Day 1–2 NA Day 3–9 BTA, Central sparing	Day 1–4 Glu↑ Day 4–12 Gln↑	< 24 h CBF↓ Day 3–5 CBF↑	[3, 6, 8, 18]
This case	4 years	F	Parainflu 3 Rhino C	+	+	-	NA	Day 8 Gln↑	33 h Central sparing, CBF↑	

*AESD* Acute encephalopathy with biphasic seizures and late reduced diffusion, *ASL* Arterial spin labeling, *BTA* Bright tree appearance, *CBF* Cereberal blood flow, *CFS* Complex febrile seizure, *F* Female, *Flu* Influenza, *Gln* Glutamine, *Glu* Glutamate, *HHV-6/7* Human herpesvirus 6/7, *IC* Impaired consciousness, *M*, Male, *MEEX* Mild encephalopathy associated with excitotoxicity, *MRS* MR spectroscopy, *NA* No abnormalities, *Parainflu*, Parainfluenza, *RSV* respiratory syncytial virus, *SE* Status epilepticus, *UK* Unknown

Considering that MEEX and AESD share the same pathophysiology, we assume that an MRI for patients with MEEX should be performed ≥ 8 h after the early seizure as well as AESD. Further research is necessary to prove the above hypothesis. Although we diagnosed our patient with MEEX based on the clinical course and findings of MRI and MRS, ASL in our patient also showed a central sparing pattern similar to that in AESD. This is the first case confirming the usefulness of ASL in the diagnosis of MEEX

In conclusion, considering that AESD and MEEX are within the same spectrum of encephalopathy associated with excitotoxicity, ASL may be useful in the early diagnosis of MEEX as well as AESD, allowing for the initiation of early intervention.

## Data Availability

All data generated or analyzed during this study are included in this published article.

## Abbreviations

AESD: Acute encephalopathy with biphasic seizures and late reduced diffusion
MEEX: Mild encephalopathy associated with excitotoxicity
BTA: Bright tree appearance
MRI: Magnetic resonance imaging
MRS: Magnetic resonance spectroscopy
ASL: Arterial spin labeling
CBF: Cerebral blood flow
DWI: Diffusion-weighted imaging
ADC: Apparent diffusion coefficient
EEG: Electroencephalogram
